# The management of critical bone defects: outcomes of a systematic approach

**DOI:** 10.1007/s00590-024-04050-1

**Published:** 2024-08-02

**Authors:** Shao-Ting Jerry Tsang, Adrian Jansen van Rensburg, Jason van Heerden, Gadi Zwe Epstein, Rudolph Venter, Nando Ferreira

**Affiliations:** Division of Orthopaedic Surgery, Department of Surgical Sciences, Faculty of Medicine and Health Sciences, Stellenbosch University, Cape Town, 7505 South Africa

**Keywords:** Open fracture, Critical bone defects, Reconstruction techniques

## Abstract

**Background:**

The reconstruction of segmental long bone defects remains one of ‘The holy grails of orthopaedics’. The optimal treatment of which remains a topic of great debate. This study aimed to evaluate the outcomes following the management of critical-sized bone defects using a classification-based treatment algorithm.

**Methods:**

A retrospective review of all patients undergoing treatment for segmental diaphyseal defects of long bones at a tertiary-level limb reconstruction unit between January 2016 and December 2021, was performed. The management of the bone defect was standardised as per the classification by Ferreira and Tanwar (2020).

**Results:**

A total of 96 patients (mean age 39.8, SD 15.2) with a minimum six months follow-up were included. Most bone defects were the result of open fractures (75/96) with 67% associated with Gustilo-Anderson IIIB injuries. There was a statistical difference in the likelihood of union between treatment strategies with more than 90% of cases undergoing acute shortening and bone transport achieving union and only 72% of cases undergoing the induced membrane technique consolidating (*p* = 0.049). Of those defects that consolidated, there was no difference in the time to bone union between strategies (*p* = 0.308) with an overall median time to union 8.33 months (95% CI 7.4 – 9.2 months). The induced membrane technique was associated with a 40% risk of sepsis.

**Conclusion:**

This study reported the outcomes of a standardised approach to the management of critical-sized bone defects. Whilst overall results were supportive of this approach, the outcomes associated with the induced membrane technique require further refinement of its indications in the management of critical-sized bone defects.

**Level of evidence:**

4.

## Introduction

The reconstruction of segmental long bone defects remains one of ‘The holy grails of orthopaedics’. [1] The presence of a segmental defect is associated with prolonged treatment and compromised outcomes following trauma [2, 3] and fracture-related infection [4]. One of the most significant advances in this field has been the gradual move towards an accepted definition of “critical-sized” bone defect, with much of this progress due to secondary analysis of the SPRINT trial [5]. At present, defects larger than 2 cm in length and with more than 50% circumferential bone loss are considered critical bone defects and unlikely to heal without further intervention [3]. Despite this progress, there remains equipoise within the orthopaedic community regarding the optimal treatment strategy [1]. Strategies which all require a significant allocation of resources, including time, at the cost to both patient and health care providers [6].

Ferreira and Tanwar recently proposed a classification system and treatment algorithm in the management of post-traumatic critical-sized bone defects. In this system consideration is given to the following; the size of the bone defect (< 2 cm, 2 – 6 cm, 6 – 12 cm, or > 12 cm), soft tissue quality (no deficit, defect requiring reconstruction, or unreconstructable defect), and host type (no compromise, local or systemic compromise, or treatment would be worse than the disease for the patient) [6]. It was proposed that treatment strategies should be tailored to address all the elements identified and stratified by the classification system [6].

This study aimed to evaluate the outcomes following the management of critical-sized bone defects using a classification-based treatment algorithm.

## Methods

This single-centre retrospective study was performed at a tertiary-level limb reconstruction unit. The records of all patients undergoing treatment for segmental diaphyseal defects of long bones between January 2016 and December 2021 were retrospectively reviewed. Patient demographics, co-morbidities, aetiology and site of defect, defect management technique, follow-up period, and outcomes (clinical and radiological) were collected.

A critical bone defect was defined as a bone defect that will not heal if left untreated, with segmental defects of more than 60mm generally regarded as large defects [2, 5, 7]. Non-union was defined as fractures that failed to unite nine months after the injury or showed no radiological progression to union in three consecutive months. Treatment success was defined as bone union.

Chronic osteomyelitis was defined as an infection of the bone with associated necrosis with a duration of at least 10 days, where the pathogens were thought to have resisted either intracellularly or interstitially in biofilm-or persister-states [8]. The diagnosis of fracture-related infection was made according to the international consensus definition proposed by Metsemaker et al. and modified by Govaert et al. in 2020 [9, 10].

Patients with periarticular bone defects, tumour-related bone defects, or fewer than six months of follow-up were excluded.

The bone defect treatment algorithm used in this present study, depending on host and soft tissue grading, was as described by Ferreira and Tanwar [6]. In brief;

- <20 mm defect: Acute shortening or primary bone grafting
- 20–60 mm: Induced membrane or acute shortening and lengthening or bone transport
- 60–120 mm: Bone transport
- >120 mm: Bone transport

Statistical analysis was performed using SPSS v25 (IBM Corp, Armonk, NY, USA). Parametric data are reported as mean and standard deviation (SD) with 95% confidence intervals (CI) where appropriate. Non-parametric data are described with median, interquartile range and range. Categorical data are described as frequencies and/or counts, with 95% CI where appropriate. Depending on the distribution, associations were investigated using a one-way analysis of variance (ANOVA) or a Mann-Whitney U/Median test. Pearson Chi-squared test or Fisher’s Exact test, where appropriate was used to detect significant differences between categorical data. Estimation of time to bone union was performed using Kaplan-Meier statistics, and comparisons were made using Log-rank analysis.

## Results

**Fig. 1:**
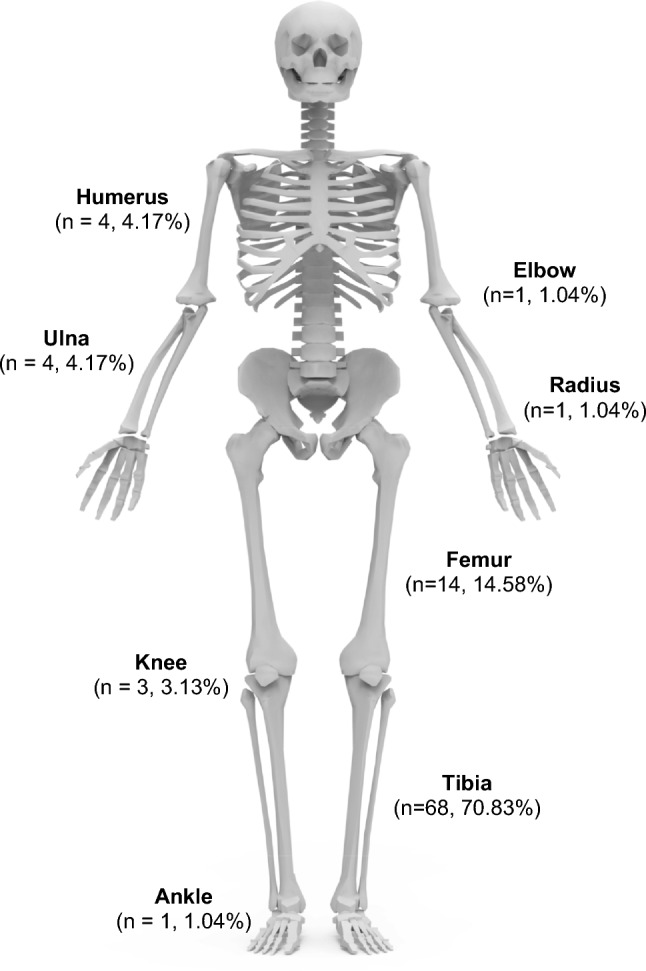
Anatomical distribution of critical bone defects

The final cohort comprised 96 patients (63 males and 33 females) with a mean age of 39.8 (SD 15.2, range 10–82) years. Patient demographics, co-morbidities, and aetiologies of the bone defects are shown in Table 1. Thirty-four patients (35%) were classified as A hosts, while 55 (65%) were classified as B hosts; no C hosts underwent bone defect reconstruction in the current cohort. The majority (75/96, 78.1%) of segmental bone defects were a result of open fractures with 50/75 (66.7%) being Gustilo-Anderson IIIB injuries. Soft tissue defects requiring reconstruction (β soft tissue defect) were found in 50 patients (52%) while the remaining patients did not require any soft tissue reconstruction (⍺ soft tissue). The most affected bone was the tibia (70.8%). The full distribution of affected anatomical sites is shown in Fig. 1.

**Table 1:** Patient demographics, co-morbidities, and aetiologies of bone defect

	*n*=96
Male gender (%,*n*)	65.63% (63)
Age (Mean, SD)	39.8 (±15.2)
Smoking (%,*n*)	51.04% (49)
HIV (%,*n*)	12.50% (12)
Diabetes (%,*n*)	5.21% (5)
Aetiology (%,*n*)	
*Pedestrian vehicle accident*	34.38% (33)
*Motor vehicle accident*	20.83% (20)
*Fracture-related infection*	13.54% (13)
*Gun-shot wound*	7.29% (7)
*Chronic osteomyelitis*	6.25% (6)
*Assault*	3.13% (3)
Injury type (%,*n*)	
*Open*	78.13% (75)
*Closed*	21.88% (21)
Gustillo-Anderson classification (%) (*n*=75)	
*I*	1.33% (1)
*II*	10.67% (8)
*IIIA*	20.00% (15)
*IIIB*	66.67% (50)
*IIIC*	1.33% (1)

Data reported as mean ± standard deviation, median (interquartile range) or as frequencies with counts in parenthesis.

Bone defect reconstruction strategies included acute shortening ± subsequent lengthening in 34 patients (35%), bone transport in 32 patients (33%), the induced membrane technique in 25 patients (26%) and custom-made intercalary grafts in five patients (5%). The employed treatment strategy according to bone defect size, soft tissue defect type and host category are shown in Table 2. The five patients who underwent intercalary prosthesis reconstruction of their bone defects were excluded from the union analysis. There was a statistically significant difference in the likelihood of achieving bone union between management strategies (*p* = 0.049) (Table 3). The lowest likelihood of union was amongst those treated with the induced membrane technique (18/25, 72.0%). There was no statistically significant difference in the likelihood of union between bone transport (29/32 (90.1%)) and acute shortening (32/34, 94.1%) (*p* = 0.471).

**Table 2:** Treatment according to Ferreira and Tanwar (2020) classification

Soft tissue defect type	Bone defect type				Host category
	I < 2 cm	II 2 – 6 cm	III 6–12 cm	IV > 12 cm	
⍺	*n*=4 Shortening (4)	*n*=12 Induced membrane (6) Bone transport (2) Shortening (3) Shorten + Lengthen (1)	*n*=2 Bone transport (1) Shorten + Lengthen (1)	*n*=5 Bone transport (2) Induced membrane (1) Intercalary prosthesis (2)	A
	*n*=5 Shortening (5)	*n*=11 Induced membrane (4) Bone transport (2) Shortening (4) Shorten + Lengthen (1)	*n*=4 Bone transport (1) Shorten + Lengthen (2) Intercalary prosthesis (1)	*n*=3 Bone transport (1) Intercalary prosthesis (2)	B
β	*n*=1 Shortening (1)	*n*=10 Induced membrane (2) Bone transport (5) Shortening (3)	*n*=1 Bone transport (1)	*n*=0	A
	*n*=6 Shortening (6)	n=30 Induced membrane (12) Bone transport (15 Shortening (3)	n=1 Bone transport (1)	*n*=1 Bone transport (1)	B
γ	*n*=0	*n*=0	*n*=0	*n*=0	A
	*n*=0	*n*=0	*n*=0	*n*=0	B

**Table 3:** Outcomes of reconstruction techniques used for critical bone defect management

	Acute shortening	Bone transport	Induced membrane	*p* Value
*N* = 91	*n* = 34	*n* = 32	*n* = 25	
Host status				
*A*	38% (13)	34% (11)	36% (9)	
*B*	62% (21)	66% (21)	64% (16)	
*C*	0% (0)	0% (0)	0% (0)	
Soft tissue status				
*⍺*	62% (21)	28% (9)	44% (11)	
*β*	38% (13)	72% (23)	56% (14)	
*γ*	0% (0)	0% (0)	0% (0)	
Bone defect length (mean, mm) (±Standard deviation)	31.53 (± 18.12)	50.56 (± 16.49)	47.88 (± 34.84)	*p* = 0.003
Union (%, *n*) 95% CI	94.12% (32) 80.3–99.3%	90.63% (29) 75.0–98.0%	72.00% (18) 50.6–87.9%	*p* = 0.049
Sepsis (%, *n*) 95% CI	23.53% (8) 10.7–41.2%	9.38% (3) 2.0–25.0%	40.00% (10) 21.1–61.3%	*p* = 0.026
Malalignment (%, *n*) 95% CI	5.88% (2) 0.7–19.7%	18.25% (6) 7.2–36.4%	20.00% (5) 6.8–40.7%	*p* = 0.185
Limb length discrepancy (%. *n*) 95% CI	82.35% (28) 65.5–93.2%	31.25% (10) 16.1–50.0%	24.00% (6) 9.4–45.1%	*p* < 0.001
Follow-up time (median, months) 95% CI	19.0 18.6–35.7	16.0 16.6–24.1	10.0 8.24–16.8	*p* = 0.007

Date reported as number and percentage, and median plus 95% confidence intervals (CI).

**Fig. 2:**
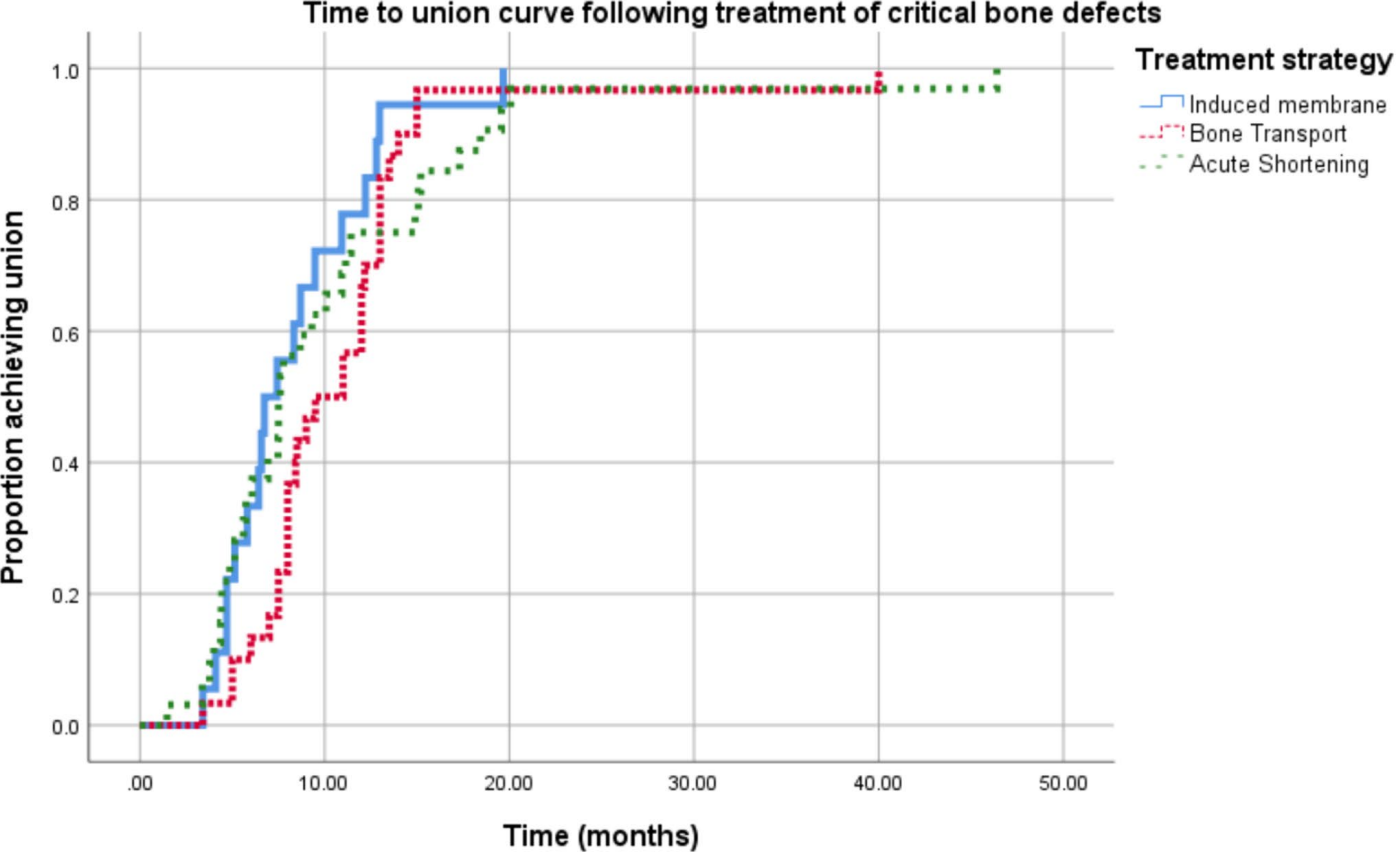
The Kaplan-Meier analysis for critical bone defects treatment strategies. Time to union curve following treatment of critical bone defects (excluding cases treated by Greenbone bone substitute and truss reconstruction)

Kaplan-Meier statistics with log-rank analysis revealed that the overall median time to bone union of the different treatment strategies was 8.33 months (95% CI 7.4 – 9.2 months). There was no statistically significant difference (*p* = 0.308) in the median time to bone union between cases managed with acute shortening (7.5 months, 95% CI 7.1 – 7.9), bone transport (9.5 months, 95% CI 6.1 – 12.9), and the induced membrane technique (6.7 months, 95% CI 4.9 – 8.5) (Fig. 2).

There was a statistically significant difference in the risk of sepsis between the treatment strategies (*p* = 0.026) with 10/25 (40.0%) cases in the induced membrane technique developing secondary infection at the site of the defect.

There was a statistically significant difference in the risk of a resultant leg length discrepancy following reconstruction between the treatment strategies (*p* < 0.05). Between-group comparisons revealed statistical differences between acute shortening (28/34, 82.4%) and all other treatment strategies (*p* < 0.05). There was no statistical difference (*p* = 0.382) between bone transport (10/32, 31.3%) and the induced membrane technique (6/25, 24.0%).

## Discussion

This study reported the outcomes following the management of critical-sized bone defects using a classification-based treatment algorithm. There was a statistical difference in the likelihood of union between treatment strategies with more than 90% of cases undergoing acute shortening and bone transport achieving union and only 72% of cases undergoing the induced membrane technique consolidating (Table 2). On analysis of all the cases that achieved consolidation, there was no difference in the time-to-bone union between strategies with an overall median time of 8.3 months (Fig. 2). The induced membrane technique was associated with a 40% risk of sepsis. Acute shortening was associated with a clinically significant leg length discrepancy in 82% of cases.

There was a significantly lower likelihood of bone union following the induced membrane technique (72%) in the treatment of critical-sized bone defects. In those that did achieve union with the Masquelet technique, the median time to union was 6.7 months. In a systematic review and meta-analysis of outcomes following the induced membrane technique for the treatment of bone defects that included 48 observational studies (1,386 cases), Fung *et al.* reported that 82% of cases achieved union after the first grafting procedure, with 87% achieving union after repeated grafting procedures. The mean time to union was 6.6 months (1.4–58.7 months) after bone grafting [11]. In addition, the meta-analysis reported an 18% risk for unplanned procedures and a 10% risk of secondary infection. A sub-analysis, using logistic regression, identified the presence of pre-operative infection as the primary risk factor for non-union of the defect with larger tibial defects being at greater risk of secondary infection [11]. A single-centre cohort study conducted after this meta-analysis also found that tibial defects following excision for osteomyelitis were at greater risk of non-union when treated with the induced membrane technique [12]. In this present study, the underlying aetiology for the bone defect was an infection in 19/96 (20%) cases with 68/96 (71%) cases located in the tibia with a mean defect size of 40.54 mm ± 21.68. Whilst the overall prevalence of secondary infection was 21/96 (22%), 10/25 (40%) cases using the induced membrane technique experienced secondary infection. Similar results have been previously reported in the literature. In a cohort of bone defects in the tibia (mean defect size 58 mm) treated with the induced membrane technique, 60% failed to unite following initial bone grafting and 40% experienced secondary infection [13]. With a significantly increased risk of non-union and secondary infection associated with the induced membrane technique, particularly with larger defects, those in the tibia, and the presence of pre-existing infection, it would be prudent to reconsider the indications for this technique. Even the original describer of the technique cautioned against its use for defects greater than 100 mm in an instructional review published by the French Society of Orthopaedic Surgery and Traumatology [14, 15].

Patients undergoing acute shortening or bone transport in this series experienced bone union in more than 90% of cases with a median time to bone union of 7.5 months and 9.5 months, respectively. The mean bone defect length in patients undergoing acute shortening and bone transport were 31.5 mm and 50.6 mm, respectively. There was a 17% risk of secondary infection and a 12% risk of malalignment associated with these techniques. Within the acute shortening group, 82% had a clinically significant limb length discrepancy for which patients were counselled pre-operatively and managed with a planned re-lengthening procedure as a second stage. Ilizarov techniques, such as acute shortening and re-lengthening or bone transport, are established treatment strategies in the management of bone defects [16]. The adaptability of circular external fixation also facilitates the simultaneous management of concomitant soft tissue defects and deformities. Aktuglu *et al*. performed a systematic review and meta-analysis of bone transport used in the treatment of critical-sized bone defects in the tibia; this meta-analysis of 619 patients (27 studies), of which 89% had concomitant infection, found that bone union was achieved in 90% (range 77–100%) cases. The mean bone defect length in the cohort was 6.5 cm and the mean external fixation time was 10.8 (range 2.5–23.2) months [17]. A further systematic review and meta-analysis of observational studies that reported the outcomes of bone transport with a circular frame in the treatment of infected non-unions of the tibia and femur (590 patients in 24 studies) estimated 97% union with a mean external fixation time of 10.7 months and external fixation index 1.7 months/cm [18]. The mean length of the bone defect in this aggregated cohort was 6.5 cm in patients with infected tibial non-unions and 8.0 cm in patients with infected femoral non-unions [18]. Sigmund *et al.* compared the outcomes of acute shortening and re-lengthening with bone transports in the management of infected segmental defects of the tibia [19]. Ultimately, there was no difference in the risk of recalcitrant infection or ongoing non-union between the two techniques. However, 15 of 27 bone transport cases required unplanned surgeries, including docking site procedures, to achieve bone union. The two groups had no difference in overall time in the fixator or the external fixator index [19]. Similar results have been reported in systematic reviews and meta-analyses that have compared the two Ilizarov reconstruction techniques. Using an aggregated cohort of 199 patients from five studies, Wen *et al* did not find any difference in the likelihood of bone union, risk of overall complications, or function results between the two techniques. However acute shortening was associated with a shorter external fixator index (standard mean difference 0.63) but increased requirement for bone grafting to achieve union [20].

This study was the first to assess the outcomes of a standardised approach to the management of critical-sized bone defects. In doing so, it highlighted shortcomings in currently used reconstruction strategies, such as the induced membrane technique. The retrospective nature of this study carries with it the expected biases associated with non-randomised comparative studies, namely selection, attrition, and confusion bias [21]. However, the standardised approach is reflective of current practice and thus allows assessment of these techniques used as they would be applied in a real-world setting, despite the methodological flaws.

## Conclusion

This study reported the outcomes of a standardised approach to the management of critical-sized bone defects. Whilst overall results were supportive of this approach, the outcomes associated with the induced membrane technique require further refinement of its indications in the management of critical-sized bone defects.

## References

[1] Tsang STJ, Ferreira N, RWSimpson AH (2022) The reconstruction of critical bone loss. Bone Joint Res. 11(6):409–12 35731230 10.1302/2046-3758.116.BJR-2022-0186 PMC9233404

[2] Schemitsch EH (2017) Size Matters: Defining Critical in Bone Defect Size! J Orthop Trauma. 31(5):S20-2 28938386 10.1097/BOT.0000000000000978

[3] Keating JF, Simpson AH, Robinson CM (2005) The management of fractures with bone loss. J Bone Joint Surg Br. 87–B(2):142–50 10.1302/0301-620X.87B2.15874 15736731

[4] Bezstarosti H, Metsemakers WJ, van Lieshout EMM, Voskamp LW, Kortram K, McNally MA et al (2021) Management of critical-sized bone defects in the treatment of fracture-related infection: a systematic review and pooled analysis. Arch Orthop Trauma Surg. 141(7):1215 32860565 10.1007/s00402-020-03525-0 PMC8215045

[5] Sanders DW, Bhandari M, Guyatt G, Heels-Ansdell D, Schemitsch EH, Swiontkowski M et al (2014) Critical-sized defect in the tibia: is it critical? Results from the sprint trial. J Orthop Trauma. 28(11):632–5 25233157 10.1097/BOT.0000000000000194

[6] Ferreira N, Tanwar YS (2020) Systematic approach to the management of post-traumatic segmental diaphyseal long bone defects: treatment algorithm and comprehensive classification system. Strat Traum Limb Reconstr. 15(2):106–16 10.5005/jp-journals-10080-1466 PMC9679593 36466309

[7] Roddy E, DeBaun MR, Daoud-Gray A, Yang YP, Gardner MJ (2018) Treatment of critical-sized bone defects: clinical and tissue engineering perspectives. Eur J Orthop Surg Traumatol. 28(3):351–62 29080923 10.1007/s00590-017-2063-0

[8] Lew DP, Waldvogel FA (1997) Osteomyelitis. N Engl J Med. 336(14):999–1007 9077380 10.1056/NEJM199704033361406

[9] Metsemakers WJ, Morgenstern M, McNally MA, Moriarty TF, McFadyen I, Scarborough M et al (2018) Fracture-related infection: a consensus on definition from an international expert group. Injury. 49(3):505–10 28867644 10.1016/j.injury.2017.08.040

[10] Govaert GAM, Kuehl R, Atkins BL, Trampuz A, Morgenstern M, Obremskey WT et al (2020) Diagnosing fracture-related infection: current concepts and recommendations. J Orthop Trauma. 34(1):8 31855973 10.1097/BOT.0000000000001614 PMC6903359

[11] Fung B, Hoit G, Schemitsch E, Godbout C, Nauth A (2020) The induced membrane technique for the management of long bone defects. Bone Joint J. 102(12):1723–34 33249891 10.1302/0301-620X.102B12.BJJ-2020-1125.R1

[12] Wang X, Wang S, Xu J, Sun D, Shen J, Xie Z (2021) Antibiotic cement plate composite structure internal fixation after debridement of bone infection. Sci Rep. 11(1):1–6 34413456 10.1038/s41598-021-96522-1 PMC8377006

[13] Morris R, Hossain M, Evans A, Pallister I (2017) Induced membrane technique for treating tibial defects gives mixed results. Bone Joint J. 99–B(5):680–5 28455479 10.1302/0301-620X.99B5.BJJ-2016-0694.R2

[14] Karger C, Kishi T, Schneider L, Fitoussi F, Masquelet A-CC (2012) Treatment of posttraumatic bone defects by the induced membrane technique. Orthop Traumatol Surg Res. 98(1):97–102 22244249 10.1016/j.otsr.2011.11.001

[15] Rigal S, Merloz P, Le Nen D, Mathevon H, Masquelet AC (2012) Bone transport techniques in posttraumatic bone defects. Orthop Traumatol Surg Res. 98(1):103–8 22257763 10.1016/j.otsr.2011.11.002

[16] Ilizarov GA (1989) The tension-stress effect on the genesis and growth of tissues. Part I. the influence of stability of fixation and soft-tissue preservation. Clin Orthop Relat Res. 238:249–81 10.1097/00003086-198901000-00038 2910611

[17] Aktuglu K, Erol K, Vahabi A (2019) Ilizarov bone transport and treatment of critical-sized tibial bone defects: a narrative review. J Orthop Traumatol. 20:1–14 10.1186/s10195-019-0527-1 PMC6468024 30993461

[18] Yin P, Ji Q, Li T, Li J, Li Z, Liu J et al (2015) A systematic review and meta-analysis of ilizarov methods in the treatment of infected nonunion of tibia and femur. PLoS One. 10(11):e0141973 26529606 10.1371/journal.pone.0141973 PMC4631548

[19] Sigmund IK, Ferguson J, Govaert GAM, Stubbs D, McNally MA (2020) Comparison of Ilizarov Bifocal, Acute Shortening and Relengthening with Bone Transport in the Treatment of Infected, Segmental Defects of the Tibia. J Clin Med. 9(2):279 32012855 10.3390/jcm9020279 PMC7074086

[20] Wen H, Zhu S, Li C, Xu Y (2020) Bone transport versus acute shortening for the management of infected tibial bone defects: a meta-analysis. BMC Musculoskelet Disord. 21:1–9 10.1186/s12891-020-3114-y PMC7006089 32028924

[21] Ramirez-Santana M (2018) Limitations and Biases in Cohort Studies. In: Mauricio Barría R (ed) Cohort Studies in Health Sciences. InTech, London

